# Older adults' experiences of wellbeing during the COVID-19 pandemic: a comparative qualitative study in Italy and Switzerland

**DOI:** 10.3389/fsoc.2024.1243760

**Published:** 2024-05-01

**Authors:** Iuna Dones, Ruxandra Oana Ciobanu

**Affiliations:** ^1^Faculty of Social Sciences, University of Geneva, Geneva, Switzerland; ^2^Swiss Center of Expertise in Life Course Research (LIVES), Geneva, Switzerland; ^3^Faculty of Social Work, University of Applied Sciences and Arts Western Switzerland (HETSL/HES-SO), Lausanne, Switzerland

**Keywords:** migration, coping, subjective wellbeing, health crisis, vulnerability

## Abstract

**Background:**

Particularly at the beginning of the pandemic, adults aged 65 and older were portrayed as a homogeneously vulnerable population due to the elevated health risks associated with contracting the COVID-19 disease. This portrayal, combined with travel restrictions, closures of economic sectors, country-wide lockdowns, and suggestions by governmental authorities to limit social contact, had important implications for the wellbeing of older individuals. However, older adults are a heterogeneous population who relies on different resources to cope with stressful periods, like the COVID-19 pandemic. Simultaneously, countries also employed different measures to contain the virus. Research thus far has focused on the short-term consequences of the pandemic, but studies have yet to address its long-term consequences.

**Objectives:**

We explore older adults' lived experiences nearly 2 years after the pandemic onset. Moreover, we focus on the bordering countries of Switzerland and Italy, who employed contrasting containment measures. This paper analyzes (1) How the COVID-19 pandemic impacted the experiences of wellbeing of older adults in these regions and (2) How older adults coped with the stressors brought about by the pandemic, in particular social distancing.

**Methods:**

The paper draws on 31 semi-structured interviews with 11 Swiss natives residing in Switzerland, 10 Italian migrants residing in Switzerland, and 10 Italian natives residing in Italy. Interviews were conducted from December 2021 to March 2022.

**Results:**

Coping mechanisms of the three groups related to acceptance, hobbies, cognitive reframing, telephone use, vaccine use and social distancing. However, results show heterogeneous experiences of wellbeing, with Swiss natives sharing more positive narratives than the other two groups. Moreover, Italian migrants and Italian natives expressed the long-term negative consequences of the pandemic on their experienced wellbeing.

## 1 Introduction

In March of 2020, the WHO declared COVID-19 a pandemic, making it an international public health problem (WHO, 2020). Beyond physical illness, the pandemic disrupted millions of lives around the globe through closures of schools, shops, and borders; it separated individuals from friends and family, and it caused job losses and financial strain (Hiscott et al., 2020). However, in the public discourse it was especially older adults who were portrayed as a homogeneously vulnerable and frail group (Jordan et al., 2020; Ayalon et al., 2021; Maggiori et al., 2022). Indeed, older people had higher mortality rates than younger ones (Dadras et al., 2022). They also suffered from decreased physical activity due to social distancing measures, impacting their physical health (Oliveira et al., 2022). Moreover, in comparison to pre-pandemic levels, older adults reported higher rates of anxiety and depression (Webb and Chen, 2022; Segerstrom et al., 2023) and lower subjective wellbeing (Maggiori et al., 2022).

However, compared to their younger counterparts, older adults also reported less pandemic-related stress, less social isolation, less life changes (Birditt et al., 2021), and lower rates of anxiety and depression (Webb and Chen, 2022). Furthermore, older adults' resilience was shown through their ability to develop coping strategies to maintain a certain level of subjective and psychological wellbeing (Finlay et al., 2021; Fuller and Huseth-Zosel, 2021; Bustamante et al., 2022; Facal et al., 2022; Mau et al., 2022).

Nonetheless, the ability to cope with the stressors brought about by the pandemic was influenced by factors on the micro-, meso-, and macro- levels. On the micro-level, studies have revealed that characteristics like being in good health, previous experiences of adversity, stable financial status, and social networks positively affected individuals' ability to cope with the pandemic (Guzman et al., 2023), while having a migration background was associated with increased pandemic-related worry (Ludwig-Dehm et al., 2023) and loneliness (Pan et al., 2021). At the meso-level, neighborhood parks and nature were positively related to mental and physical health (Bustamante et al., 2022; Guzman et al., 2023). At the macro-level, stricter physical distancing measures mandated by governments were associated with worse mental health (Mendez-Lopez et al., 2022). The heterogeneity of older adults' characteristics, resources, and lived experiences, as well as the differences in countries' containment measures thus call for research further exploring how older adults coped with the pandemic in different contexts.

Most studies to date focus on older individuals' wellbeing during the first lockdown in the spring of 2020 (Seifert and Hassler, 2020; Cipolletta and Gris, 2021; Falvo et al., 2021; Finlay et al., 2021; Fuller and Huseth-Zosel, 2021; McKinlay et al., 2021; Whitehead and Torossian, 2021; Bustamante et al., 2022; Facal et al., 2022; Gonçalves et al., 2022; Kremers et al., 2022) or during the first year following the pandemic onset (Fiocco et al., 2021; Atzendorf and Gruber, 2022; Brooks et al., 2022; Cohn-Schwartz et al., 2022; Derrer-Merk et al., 2022a; Garner et al., 2022; Maggiori et al., 2022; Mau et al., 2022; Donizzetti and Capone, 2023). However, few studies have been published thus far addressing the second year of the pandemic and its long-term impact on older adults' wellbeing (Gallè et al., 2021; König and Isengard, 2023). Moreover, studies have analyzed the pandemic's impact on the physical health of migrants of all ages in comparison to native-born populations in Western Europe (Canevelli et al., 2020; Aldea, 2022; Khlat et al., 2022), but to the best of our knowledge, none have addressed the differences in the lived experiences of wellbeing among older migrant and native populations with a focus on coping strategies. Furthermore, there is a paucity of literature comparing the experiences of wellbeing and coping strategies between countries that implemented contrasting COVID-19 containment measures.

We aim to bridge this gap by comparing the experiences of older adults in Italy and Switzerland – countries that implemented different COVID-19 containment measures – nearly 2 years after the pandemic onset. Specifically, we study older Swiss natives residing in Switzerland, older Italian natives residing in Italy, and older Italian migrants residing in Switzerland. This allows us to explore the experiences of wellbeing of older adults who lived under strict restriction measures, namely Italian residents, to those of adults who lived under more relaxed measures, namely Swiss natives and Italian migrants living in Switzerland. Furthermore, comparing Swiss natives and Italian migrants allows us to analyze the experiences of two groups who lived the pandemic in the same context, yet who have had different life courses. More particularly, because older Italian migrants in Switzerland often have attachments to Italy and take part in transnational practices (Ludwig-Dehm et al., 2023), their inclusion in the study allows us to explore how the situation in their country of origin impacted their COVID-19 experiences from abroad.

This paper aims to analyze (1) How the COVID-19 pandemic impacted the experiences of wellbeing of older adults in Switzerland and Italy and (2) How older adults coped with the stressors brought about by the pandemic, in particular social distancing.

## 2 Contextual background: the Swiss and Italian contexts

Despite the geographical proximity between Switzerland and Italy, the two countries implemented quite different containment measures as a response to the virus.

Compared to other European countries, on average Switzerland implemented less stringent containment measures throughout the pandemic, despite being just as impacted (Pleninger et al., 2022). The first phase of the pandemic, classified by the Federal Council as an “extraordinary situation,” lasted from March 16 to June 19, 2020 (Sager and Mavrot, 2020; Maggiori et al., 2022; Pleninger et al., 2022). From March 16 until April 26, the Swiss government gradually imposed measures closing borders, canceling cultural and sports events, banning all public and private manifestations, closing schools, restaurants, bars, as well as shops and services deemed to be unessential, and banning gatherings of more than five people. Older adults in particular were advised to stay at home and to avoid in-person social interactions with members outside their household. From April 26, 2020 containment measures were slowly eased, and on June 19, 2020, the classification of the pandemic changed from “extraordinary” to “special” (Sager and Mavrot, 2020; Pleninger et al., 2022).

During the next 2 years, Switzerland saw a series of tightening and easing of containment measures, which included regulations on mandatory vaccines or COVID-19 tests to access bars and restaurants, and mandatory masks to be worn in shops and public transport. All restrictions were then lifted on April 1, 2022 (FOPH, 2022).

Throughout the pandemic up until the data collection for this article – between December 2021 and March 2022 – the Swiss government largely relied on cooperation from the public. Although in certain periods shops, restaurants, and schools were closed, Swiss residents still enjoyed a certain amount of freedom to move and have social gatherings, albeit limited. Overall, it was left up to the individuals to regulate their behaviors within certain limits.

The Italian government, on the other hand, imposed more stringent measures throughout the pandemic. Late January 2020, the government declared a national emergency, and in February 2020, Italy was the epicenter of the health crisis in Europe (Ferrante, 2022). The Italian government quickly established lockdown “red” zones in certain areas of Northern Italy, which led to the closure of schools and restrictions of movement: residents could leave their areas of residence only for necessities like work, health reasons or family emergencies, or grocery shopping. These restrictions were applied in waves to the entire country, and on March 11 the government imposed a national lockdown, also named the “stay at home” decree (Bull, 2021). This entailed closure of borders, schools, restaurants and bars, and all nonessential shops and services. Travel between regions was prohibited and residents' movement was only allowed for essential reasons. The first Italian lockdown ended on May 3, 2020, after which most shops, restaurants, bars, and services gradually reopened while maintaining COVID-19 safety protocols (Bosa et al., 2021).

During the next 2 years, Italy also experienced a series of loosening and tightening of restrictions, but these were often more stringent than the ones imposed in Switzerland. For instance, during the second wave of the pandemic, which took place in autumn of 2020 and winter of 2021, curfews from 11 pm to 5 am were mandated and restaurants and bars had to close at 6 pm. During this time, Italian residents were strongly recommended to leave their homes only for work or health reasons, and these restrictions were gradually eased by mid-2021 (SkyTG24, 2020; Bosa et al., 2021). In April 2022, Italy declared an end to the state of emergency, and thereafter lifted all restrictions (Amanto, 2022).

Both Italy and Switzerland were successful in containing the spread of the virus (Ferrante, 2022; Pleninger et al., 2022), but at what cost to people's wellbeing?

## 3 A theoretical framework to understand wellbeing and coping strategies among older adults

### 3.1 Wellbeing

Research on wellbeing largely encompasses two forms: objective wellbeing and subjective wellbeing. The first refers to objective indicators like income, health, and living conditions. The second refers to individuals' experiences of wellbeing and to their evaluations of their lives. It is often measured with indicators like positive and negative affect, happiness, life satisfaction, and satisfaction with various life domains like social relationships, financial situation, and neighborhood conditions (Bartram, 2012; Diener, 2012; Veenhoven, 2012, 2017). In this paper, we use the term wellbeing to refer to the latter concept – to individuals' subjective experiences of wellbeing.

Some objective indicators are indeed correlated to subjective indicators – being in good health, for instance is positively associated with life satisfaction (Helliwell, 2003; Deaton, 2008; Clark et al., 2018) and, to a certain extent, so is income (Clark et al., 2008; Clark, 2011; De Jong, 2015). Studies have also debated to what extent wellbeing is dependent on genes and individual personality traits (Bartels, 2015; Røysamb et al., 2018), and to what extent it is dependent on external factors like social contexts and life events (Helliwell and Putnam, 2004). But overall, the consensus is that wellbeing is influenced by both genetic and environmental characteristics (Røysamb et al., 2014; Luhmann et al., 2021).

Most studies employ a quantitative approach and explore a wide array of determinants of wellbeing, ranging from age, to health, to income and education, to relationships and divorce, to social norms and institutions, and so on (Clark et al., 2018). Qualitative studies on wellbeing are less common (Bartram, 2012), but they valuably provide information on participants' perceptions, views and beliefs that are unaffected by researchers' pre-determined ideologies (Delle Fave et al., 2011). Especially in a context like that of the COVID-19 pandemic – a disruptive process that homogeneously categorized an entire group as vulnerable and forced individuals world-wide to reorganize their lives – a qualitative approach allows for nuanced, in-depth analyses of people's experiences of wellbeing (and vulnerability).

### 3.2 Older adults' vulnerability

Independently of the pandemic context, older adults are often characterized as particularly frail and vulnerable (Fried et al., 2001; Clegg et al., 2013). This is often due the age-related decline in physiological and psychological systems, which renders this population vulnerable to falls, hospitalization, or sudden health changes triggered by minor events, and makes them more reliant on others for care (Fried et al., 2001; Clegg et al., 2013). But vulnerability in old age is not a dichotomous state of vulnerable vs. not vulnerable, as was suggested in the public discourse during the COVID-19 pandemic. According to the life-course approach employed by Spini et al. (2017), vulnerability is defined as:

“a weakening process and a lack of resources in one or more life domains that, in specific contexts, exposes individuals or groups to (1) negative consequences related to sources of stress, (2) an inability to cope effectively with stressors, and (3) an inability to recover from stressors or to take advantage of opportunities by a given deadline.” (Spini et al., 2017, p. 8)

It is a dynamic process between stress and resources that occurs at the intersection of different areas of life (like health, work, family, etc.), and on several levels (macro-, meso-, or micro-levels) throughout the life course (Spini et al., 2017). When faced with a stressful situation like the COVID-19 pandemic, individuals must rely on the resources they accumulated throughout the life course – referred to as reserves (Cullati et al., 2018) – in order to cope with life adversities. These reserves include, but are not limited to, physical and mental health, economic savings, cultural capital resulting from education, social networks, and emotional and cognitive reserves (Cullati et al., 2018). It is in times of shocks that these reserves become the most important and mediate the impact of stressors on individuals' wellbeing; it is also during these adverse periods that inequalities between individuals' reserves become the most apparent (Widmer, 2022), leading to situations of vulnerability.

In old age, physical reserves diminish, and older adults' ability to fight infectious diseases decreases, putting them in a vulnerable situation (Bajaj et al., 2021). However, physical reserves are related to events and conditions throughout the lifespan. For example, the combination of disadvantageous childhood socioeconomic conditions, coupled with adverse adult socioeconomic conditions, increase the probability of chronic health diseases (Galobardes et al., 2007). Aging adults are thus not all equally vulnerable to the risks associated with COVID-19; their vulnerability is associated to a wide variety of life-course experiences and factors, only some of which directly related to age (Oris et al., 2020; Sneed and Krendl, 2022).

### 3.3 Pandemic impact on older adults' wellbeing

In the context of the COVID-19 pandemic, researchers have studied different measures related to wellbeing, like loneliness, social isolation, worry, anxiety, and others. This section draws on the literature focusing on various experiences of wellbeing during the pandemic among older adults.

Following the implementation of virus containment and social distancing measures, many countries reported an increase in loneliness among older adults in comparison to pre-pandemic levels (Luchetti et al., 2020; Seifert and Hassler, 2020; Holaday et al., 2021; Macdonald and Hülür, 2021; Rodney et al., 2021; Van Tilburg et al., 2021; Zaninotto et al., 2022; see also literature review by Su et al., 2023). Feelings of loneliness were particularly prevalent among older adults with no children, lower-income individuals, those living alone, and those reporting depressive symptoms (Seifert and Hassler, 2020; O'Shea et al., 2021), which highlights the role of resources and reserves in mediating the pandemic's impact on wellbeing.

Furthermore, older adults in countries all around the world experienced higher levels of stress, worry, anxiety, and depression in comparison to pre-pandemic levels. These negative mental health outcomes were more common among older single adults, among older adults of lower socioeconomic groups (Kola et al., 2021; Webb and Chen, 2022; Wettstein et al., 2022a; Zaninotto et al., 2022), among those with poor self-rated health (Wettstein et al., 2022a), and among those who were already socially isolated prior to the pandemic (Macleod et al., 2021). Social isolation is correlated to declining physical and mental health, increased mortality, and lower quality of life, and the social distancing measures introduced by the pandemic exacerbated these risks (Macleod et al., 2021).

Most studies published to date, in May 2023, concentrate on the initial weeks of the pandemic and largely focus on singular countries. Atzendorf and Gruber (2022)'s research, however, focused on the weeks following the first wave, between June and August 2020 and used SHARE data to analyze the medium-term consequences of the first pandemic wave across 25 European countries and Israel. They found that older adults in countries with high death rates and stringent measures were at increased risk of feeling depressed or lonely. Similarly, Mendez-Lopez et al. (2022) used the same data and revealed that countries' greater stringency in physical distancing measures was associated with worse mental health. This is particularly pertinent for this paper, as both Italy and Switzerland were badly hit by the pandemic (Ferrante, 2022; Pleninger et al., 2022), but they differed in containment strategies: while Italian residents were severely limited in their mobility, Swiss residents benefitted from a certain amount of freedom. Atzendorf and Gruber (2022) revealed that Italian older adults reported increased feelings of loneliness and depression after the pandemic onset to a greater extent than Swiss older adults.

The only study to date analyzing older adults' wellbeing during the two years following the pandemic onset showed that most older Europeans did not feel lonely before or during the pandemic. However, for some, feelings of loneliness increased, particularly among the less educated, those living alone, and those isolated at home (König and Isengard, 2023).

Moreover, the characterization of older adults as a homogeneous, exceptionally vulnerable population (Petretto and Pili, 2020; Seifert, 2021) engendered negative self-perceptions of aging (Losada-Baltar et al., 2021; Seifert, 2021), which have been associated with loneliness and psychological distress among older adults (Losada-Baltar et al., 2021). Their homogenous representation and the resulting ageist narrative also led to feelings of anger, increased anxiety, and perceptions of loss of autonomy and individualism by this older population (Derrer-Merk et al., 2022a,b).

Although research has documented the negative impact of the pandemic on older adults, studies have also suggested that in some ways, older adults did not suffer as much as their younger counterparts, as documented in the literature review by Seckman (2023). Older adults in the United States reported less pandemic-related stress, less social isolation (Birditt et al., 2021), and greater emotional wellbeing (Carstensen et al., 2020) than younger adults. The same result was found among Chinese adults (Jiang, 2020). Similarly, in Italy older adults reported less loneliness compared to younger age groups (Luchetti et al., 2020).

Independently of the pandemic context, older migrants are more vulnerable to loneliness and social isolation due to language and cultural barriers, low social capital, and dependence on children for support (Neville et al., 2018; Sidani et al., 2022). Moreover, older migrants often occupy disadvantaged socioeconomic positions and are in worse health than natives in the host country (Bolzman and Vagni, 2018; WHO, 2018). The pandemic and the related reduced social contacts may have thus rendered older migrants particularly vulnerable to social isolation, loneliness and negative mental health outcomes (Pan et al., 2021; Sidani et al., 2022). In fact, a study on older Chinese migrants in Belgium and the Netherlands revealed that reduced social participation and financial insecurity increased migrants' loneliness levels (Pan et al., 2021).

Furthermore, migrants often engage in transnational practices, linking them in various ways to their country of origin (Ciobanu and Ludwig-Dehm, 2020). The pandemic restrictions changed some of these transnational practices through travel bans and border closures (Nehring and Hu, 2022), which may influence older migrants' wellbeing. A survey conducted within the same research project as this paper, found that Italian migrants in Switzerland reported higher levels of worry about the COVID-19 pandemic than Swiss natives, and this difference is largely explained by engagement in transnational practices (Ludwig-Dehm et al., 2023).

Despite the increasing proportion of older migrants in Europe (UNDESA, 2020), research on the impact of the pandemic on older migrants' wellbeing is scarce.

### 3.4 Coping strategies of older adults

Research has shown that aging adults are capable of adapting and coping to various events and circumstances (Klausen, 2020; Settersten et al., 2020). Coping refers to the cognitive and behavioral efforts one carries out to prevent, tolerate, or diminish certain situations (Lazarus and Folkman, 1984; Carver, 2013; Biggs et al., 2017), and studies have found that older adults are particularly able to engage in such behaviors to diminish stressors (Yancura and Aldwin, 2008; Carstensen et al., 2020). Coping strategies are often grouped into emotion-focused and problem-focused strategies (Lazarus and Folkman, 1984; Aldwin and Revenson, 1987; Biggs et al., 2017). The first refers to strategies intended to regulate one's emotional reactions to the problem, while the latter refers to behaviors and cognitions aimed at directly managing or solving a problem (Yancura and Aldwin, 2008; Biggs et al., 2017). This includes strategies aimed at avoiding thinking about the problem – like keeping oneself busy – as well as strategies aimed at finding the positive aspects of a stressful situation (Aldwin and Yancura, 2004).

Older adults' ability to engage in these strategies can be partly explained by Carstensen's (2021) Socioemotional Selectivity Theory, which posits that social and emotional goals change depending on the perception of how much time one has left to live. As one grows older or approaches the end of their life due to illnesses or frailty, goals shift and people tend to value smaller and more meaningful social networks, they tend to spend more time with close partners, and they use cognitive resources to process more positive information (Carstensen, 2021).

Another aspect related to older adults' coping abilities concerns the aforementioned reserves accumulated throughout the life-course. Accumulation of social resources, cultural and economic capital, health reserves, and the acquisition of coping skills allow older adults to endure stressful situations or, on the contrary, the lack of such reserves can penalize them (Grundy, 2006; Cullati et al., 2018; Settersten et al., 2020).

In addition to the wellbeing consequences for older adults, studies have addressed the coping mechanisms developed by this population throughout the first wave of the pandemic. In a qualitative study, Gonçalves et al. (2022) interviewed older adults in Brazil, the United States, Italy, and Portugal, and revealed that social isolation engendered feelings of restriction in terms of interaction with friends and family and ability to participate in leisure activities. At the same time, older adults were also able to cope with the situation by dedicating their time to hobbies, using technological resources to stay close to friends and family, or involving themselves in religious and spiritual activities. Despite the different cultures and contexts of this study's participants, researchers found homogeneity in their coping mechanisms. Several studies confirmed these findings with different samples of older U.S. American adults (Finlay et al., 2021; Fuller and Huseth-Zosel, 2021; Whitehead and Torossian, 2021), and Bustamante et al. (2022) revealed that time spent in parks and outdoor spaces boosted physical, mental, and social wellbeing.

Similarly, Mau et al. (2022) found that for older Danish adults, adapting to the situation by reframing their mindset, finding ways to maintain social contacts and a sense of community, and staying active were important coping behaviors that helped them maintain a good level of wellbeing. In Italy, older adults experienced the first pandemic wave in heterogeneous ways: those who felt alone pre-pandemic expressed that isolation had a negative impact on their wellbeing. Others were able to cope with the situation by exploring hobbies and maintaining contacts with friends and family through telephone use (Cipolletta and Gris, 2021).

However, the only study on older migrants' wellbeing and coping strategies by Pan et al. (2021) found that neither problem-focused coping strategies, nor emotion-focused coping protected against increased loneliness during the pandemic.

These studies reveal that, at least for the first half of 2020, older adults employed coping mechanisms to endure the pandemic, but we still know little of their experiences after the first COVID-19 wave. A longitudinal qualitative study on Canadian older persons explored their experiences over a 10-month period from May 2020 to February 2021 (Brooks et al., 2022). It found that the longevity of pandemic restrictions was partially responsible for older adults' declines in wellbeing. Simultaneously, participants used similar coping mechanism employed during the first pandemic wave to maintain their wellbeing: they stayed active, found ways to stay in contact with friends and family, and adopted positive mindsets.

Nonetheless, cross-country research on the experiences of wellbeing among older adults, and more particularly in the years following the pandemic onset, is still scarce. We therefore aim to bridge this gap by exploring the lived experiences and coping mechanisms of older individuals in two countries that had contrasting COVID-19 containment measures like Italy and Switzerland. Furthermore, we analyze how having connections to both countries, as is the case of Italian migrants in Switzerland, influences the lived experiences of these individuals.

## 4 Data and methods

Our study focuses on three groups of older adults (65+): (1) Swiss natives, defined as individuals who were born in Switzerland and whose parents were also born in Switzerland, (2) Italian international migrants from the south of Italy, defined as individuals who were born in southern Italy, whose parents were also born in Italy, and who migrated to Switzerland, and (3) Italian natives, defined as those who were born in the south of Italy, resided in the south of Italy at the time of the research, and whose parents were also born in Italy. There are several reasons for the inclusion of these specific groups in our study. First, Italians constitute one of the largest cohorts of foreign nationals aged 65 and above residing in Switzerland (FSO, 2020). Second, a significant part of older Italians migrated to Switzerland between the 1950s and 1970s, with the majority originating from economically disadvantaged regions of Southern Italy (Wessendorf, 2007). They primarily migrated for financial reasons or to reunite with family who had relocated as labor migrants (Bolzman et al., 2004; Riaño and Wastl-Walter, 2006), and we therefore analyze older adults with a very specific migration background. Third, by comparing migrants from Southern Italy to natives from the same regions, we can explore the lived experiences of individuals who were raised in similar social contexts.

The sample for this paper is derived from an original quantitative survey conducted between June and November 2020 in the project TransAge: “Transnational aging among older migrants and natives: A strategy to overcome vulnerability.” Respondents to the qualitative interviews had already participated to the TransAge survey and had agreed to be further contacted for a follow-up interview. In total, 31 individuals participated to the study, of which 11 were Swiss natives, 10 were Italian migrants residing in Switzerland, and 10 were Italian natives residing in Italy.

To ensure diversity of wellbeing experiences among each of the three groups, we attempted to recruit individuals with low and high levels of life satisfaction. To do so, we based ourselves on Diener's Satisfaction with Life Scale (Diener et al., 1985), included in the TransAge questionnaire. More specifically, we focused on the scale item “I am satisfied with my life.” In the survey, participants were asked to indicate the strength of their agreement with this statement on a scale ranging from 1 (strongly agree) to 7 (strongly disagree). We thus contacted a roughly equal number of participants who stated being satisfied with their lives (scores 6 or 7) and participants who were less satisfied (scores 5 or less). Simultaneously, we checked the general life satisfaction scores drawing on the 5-item scale to assure coherence between the single-item and the total score (Diener et al., 1985).

The first author conducted semi-structured one-to-one interviews with the 31 community-dwelling older adults between December 2021 and March 2022, during the fifth wave of COVID-19, when social distancing was still strongly advised. Consequently, all interviews were done by telephone,^1^ except for one participant who preferred to meet in person. Participation in the study was voluntary, and all participants gave oral consent to be interviewed and recorded. Interviews lasted an average of 45 min, and they were conducted in French or Italian. They were audio-recorded and subsequently transcribed verbatim and anonymized. Participant quotes in this paper were translated into English by the first author, and every participant was given a pseudonym.

Participants were asked open-ended questions that prompted them to reflect on their experiences throughout the pandemic. First, they were asked to describe their feelings at the beginning of the pandemic, any impact that the confinement period had on their wellbeing, on their social habits, oron their daily lives. They were also encouraged to share how they coped with this period. They were then asked to reflect on the years after the onset of the sanitary crisis and describe any difficulties they faced and any strategies used to surmount these difficulties. Participants were also invited to share what their daily and social lives looked like at the time of interview, and how they felt about any long-lasting changes they may have experienced.

Interviews were analyzed using an inductive thematic analysis using qualitative coding software NVivo. The study was approved by the Ethics Committee of the Faculty of Social Sciences of the University of Geneva.

## 5 Results

### 5.1 Sample description

The 11 Swiss natives and 10 Italian migrants resided in the Swiss cantons of Geneva, Vaud, or Ticino, while the 10 Italian natives resided in the Italian regions of Sicily, Apulia, Sardinia, Abruzzo, Basilicata, or Campania. Participant characteristics by group are shown in Table 1. In comparison to the larger TransAge quantitative study, there is an over-representation of participants with medium and higher level of education among Italian migrants and natives, which will be taken into consideration in the discussion of the results.

**Table 1:** Sample charactertistics.

**Descriptive variable**	**Swiss natives Mean or n**	**Italian migrants Mean or n**	**Italian natives Mean or n**
	***n*** = **11**	***n*** = **10**	***n*** = **10**
Age	74.6	73.9	78.1
Satisfaction with life item^a^	5.7	4.7	6.1
**Sex**			
Male	7	7	5
Female	4	3	5
**Relationship status**			
Married or in a relationship	9	8	7
Widowed and/or single	2	3	3
**Living arrangement**			
Alone	1	3	3
Spouse and/or children	10	7	7
**Family situation**			
With children	8	10	10
Without children	3	0	0
**Education level** ^b^			
Low	0	4	4
Medium	5	3	3
High	6	3	3
**Making ends meet** ^c^			
Easy	6	4	6
Neither / Difficult	5	6	4

^a^The means of the Satisfaction with life item are calculated from the levels of life satisfaction at time of interview. ^b^Low level of education corresponds to those whose highest level of education is lower secondary school. Medium education level corresponds to those who completed vocational or training school, or higher secondary school. High level of education corresponds to those with advanced technical degree or university degree. ^c^Making ends meet measures how difficult or easy it is for the participant and their household to make ends meet.

### 5.2 Comparative accounts of wellbeing in times of pandemic

When recounting their experiences throughout the first 2 years of the pandemic in Switzerland and Italy, participants across the three groups coupled their narratives, whether positive or negative, with coping strategies they employed to manage the impact of the pandemic on their wellbeing. The themes that we identified correspond to emotion-focused coping and problem-focused coping strategies documented in the coping literature (Lazarus and Folkman, 1984; Aldwin and Revenson, 1987; Biggs et al., 2017). Emotion-focused coping refers to strategies aimed at regulating the emotions that arise because of a stressful situation, which also includes engagement in activities as a way to distract oneself. Problem-focused coping, on the other hand, refers to behaviors and cognitions targeted toward solving or managing a problem (Yancura and Aldwin, 2008). Strategies like social contact through telephone use involves elements of both emotion-focused and problem-focused coping. It refers to emotional support received by friends and family, it can entail concrete help in understanding how to confront an adverse situation, and it is a strategy directed at compensating for decreased in-person contact (Aldwin and Yancura, 2004).

Table 2 shows the behaviors adopted by participants that correspond to these two overarching coping mechanisms. We found that certain strategies adopted during the first lockdown were no longer used at the time of interview. Thus, in Table 2, we list the themes found in the data by pandemic period.

**Table 2:** Coping strategies used at pandemic onset and at time of interview.

**First lockdown: 2020**		**Time of interview: December 2021–February 2022**	
**Emotion-focused coping**	**Problem-focused coping**	**Emotion-focused coping**	**Problem-focused coping**
- Acceptance - Keeping busy/Hobbies - Nature - Cognitive reframing: “finding the silver lining” - Social contact through telephone use	- Social distancing measures - Social contact through telephone use	- Social contact through telephone use	- Social distancing measures - Vaccine - Social contact through telephone use

Source: Own elaboration by the authors.

During the first months of the pandemic, the primary emotion-focused strategies adopted by older adults in our sample related to acceptance of the health crisis, keeping busy through hobbies and exercise, appreciation of the natural environment, and attitudes aimed at “finding the silver lining,” which involves strategies aimed at trying to find the positive aspects of the problem at hand, and which the literature often refers to as cognitive reframing (Aldwin and Yancura, 2004; Robson and Troutman-Jordan, 2014). In terms of problem-focused coping, participants evoked the importance of social distancing measures both during the initial lockdown and at the time of interview, and many later relied on vaccines as a mean to decrease the probability of severe illness.

The subsequent sections are organized as follows: First, we detail, by group, participants' experiences of wellbeing during the first lockdown and the coping strategies they adopted to face this period. Then, we analyze how social distancing measures and decreased social contacts impacted participants in each of the three groups, and we outline participants' social habits and coping strategies at the time of interview.

#### 5.2.1 Wellbeing during the first lockdown

Although participants in all three groups used similar coping strategies throughout the pandemic, their narratives of wellbeing differed.

##### 5.2.1.1 Experiences of Swiss natives

All Swiss native older adults, except for one, described the first confinement period in positive terms and expressed not having been particularly bothered by it. They often associated their wellbeing to being able to keep busy through various hobbies and interests, and by enjoying the natural landscapes around them, as indicated in the following excerpts:

“I think I was very relaxed…I have so many books at home...I have the watercolors, I have so many things to do here, creatively, with my hands or with my head, it doesn't bother me, so...the confinement didn't bother me at all.” (Irène, 77, F, Swiss native)

“So, at home, my wife plays the piano. She has a gentleman who comes to the house. Oh yeah, she hasn't had a lesson in a year at home, but she took lessons with Zoom. You know how it is. So, she has a lot of work, piano homework. I did a little bit of crafting. I did a little bit of Spanish with French-Spanish classes.” (Nicolas, 71, M, Swiss native)

“We remained a little locked up. But we had…it was a beautiful weather. There was the spring and everything, everything was beautiful. We enjoyed our patio. We got back to reading. We did a lot of stuff like that.” (Lydia, 79, F, Swiss native)

Some Swiss participants mentioned increased telephone use to share moments with friends and family. Others described their wellbeing by comparing themselves to others, thus engaging in cognitive strategies to frame their attitude and outlook on the situation. François, for instance, often spends part of the year in Barcelona, and when talking about his wellbeing, he compares the Swiss restrictions to those of Barcelona. He elaborates:

“We were very lucky because we weren't confined like…in Barcelona. In Switzerland, that wasn't the case. Of course, there were things we couldn't do any more, but there was still a lot for us to do. We could take the car, we could go for a walk. Well, the borders were closed. Well, we didn't suffer, my wife and I…our sons either.” (François, 81, M, Swiss native)

When reflecting on the virus-containment measures, others simply stated that they just had to accept the situation and adapt their behaviors accordingly. Pierre, for example, states:

“You have to adapt. We adapt by respecting the rules, not like people who cheat [by not following the rules]. We respect the rules, but we adapt.” (Pierre, 71, M, Swiss native)

Overall, the first months of the pandemic were described in positive terms by most of Swiss older adults. Most of them portrayed themselves as being in good health and they did not evoke fears related to the virus. However, one Swiss participant expressed the negative impact of this period on his wellbeing. He recounts:

“[We lived this period] quite badly because we were old, very old. The Ticino police chief was more or less telling everybody to put us in the freezer. I mean, not quite like that…he made a statement that caused quite a stir…[The situation] was not very conducive to being cheerful, let's say.” (Gianni, 88, M, Swiss Native)

For Gianni, the government lockdown meant being “stuck at home,” as he says, and relying on institutional support. His quote shows the way he experienced the confinement measures and the public discourse as an older-old person.

##### 5.2.1.2 Experiences of Italian migrants in Switzerland

Similarly to Swiss natives, Italian migrants residing in Switzerland used cognitive strategies to frame the lockdown's impact on their wellbeing. They, too, evoked Switzerland's lenient containment measures as an important aspect that helped them surmount this period, particularly in terms of the freedom it gave them to spend time in nature. Giulia, for example, explains:

“Here in Switzerland, here in Geneva, I didn't feel this need for freedom like in other countries. For me, we were free here. I live near a park, I could take my walk every day. I have a small but very nice little apartment that has visibility on both sides, left and right, so I didn't feel like I was in prison.” (Giulia, 70, F, Italian migrant)

Italian migrants also turned to activities like reading, taking walks, and exercising to keep themselves busy during this period. However, although they lived the pandemic in the same context as the Swiss natives, there was more heterogeneity in Italian migrants' narratives of this containment period. While most stated that they simply accepted the situation and the lockdown did not negatively impact their wellbeing, some expressed feelings of loneliness and isolation. Gabriele (69, M), for example, says he felt isolated from the outside world at the beginning of the pandemic, he described his life during this period as monotonous. However, he kept himself busy by going on walks and exercising.

Others tried to overcome their feelings of loneliness by staying in communication with family, but it was not always helpful. When asked about any difficulties he faced during the lockdown, Alberto explains:

“A little bit of loneliness and missing family, that's it. It weighed on me a little bit. We used to phone my children, but no luck. My children also suffered; my youngest daughter suffered a lot and now we slowly recover.” (Alberto, 77, M, Italian migrant)

Italian migrants in Switzerland still hold transnational ties to their country of origin; a quantitative analysis of the TransAge survey found a higher level of worry about the pandemic among Italian migrants in Switzerland in comparison to Swiss natives (Ludwig-Dehm et al., 2023). We were therefore interested in investigating whether Italian migrants evoked the COVID-19 situation in Italy when describing their own experiences of wellbeing, but none of our participants organically elicited Italy's situation in their narratives. We subsequently asked participants whether they were impacted in any way by the pandemic in Italy, and responses were heterogeneous. A large part expressed not having been impacted at all, others stated that they were sorry for the high numbers of deaths in Italy and they kept in contact with family, but were not particularly affected. Few of our participants, however, disclosed the emotional suffering they experienced due to Italy's high death rates, as demonstrated by the following quotes:

“I felt tremendous suffering […] I followed a lot, every day I was watching the Italian news. And it was, for me it was just – I don't want to say worse than the war, it was a virtual war, people dying without weapons, people dying without the bombs, without being machine-gunned, but they were dying like flies.” (Giulia, 70, F, Italian migrant)

“Terrible, I felt really bad, I mean I don't know why we got to that point.” (Sara, 78, F, Italian migrant)

Although Italian migrants and Swiss natives lived the pandemic in the same context and both used similar coping strategies during the first confinement period, interviews show that Italian migrants' experiences were slightly more heterogeneous than Swiss natives', with a few migrants expressing feelings of loneliness and emotional anguish, emotions that were absent in Swiss natives' accounts.

##### 5.2.1.3 Experiences of Italian natives

In comparison to Swiss natives and Italian migrants in Switzerland, most Italian natives residing in Italy expressed feelings of worry, sadness, and fear when recounting their lockdown experiences, but most of them coupled their hardships with feelings of acceptance. Tommaso, for example, recounts:

“To hear on television, from the media, that there are deaths and deaths and deaths, obviously the concern is there. The fear, the terror even, of suffering these negative effects.” (Tommaso 84, M, Italian native)

But later, when discussing the lockdown, he continues:

“I stayed peacefully at home with a nice long beard, growing it out. I accepted it, though, because those were the rules. You had to accept them.” (Tommaso, 84, M, Italian native)

Similarly, Paolino couples the dismay brought on by the pandemic lockdown with feelings of acceptance, as well as behaviors aimed at avoiding contagion. He explains:

“The beginning of the pandemic I accepted it begrudgingly, at home, and I stayed at home despite my habits, because having lived a life always on the move – until now I was always around. That thing, the pandemic, I accepted it, and for 3 months I stayed at home, I would only go get some groceries, the bare minimum.” (Paolino, 86, M, Italian native)

In contrast to Swiss natives and Italian migrants, few Italian natives mentioned having turned to hobbies to fill up their time during the first lockdown. Some mentioned the importance of spending time outside, of having a balcony or a garden. Most of them cited phoning friends and family for emotional support, to pass time, and to update each other on their health, and most declared having used the phone for communication more than pre-COVID times. To respect social distancing rules, one participant even used intercom to communicate with family in the same building; she says:

“We used to talk to each other by intercom and by phone, we all live in the same building, so by intercom, by phone we used to talk to each other, and then if somebody went out, they would walk by the kitchen door, which was made of glass, and then we would see each other.” (Rosa, 71, F, Italian native)

Despite the coping strategies employed by Italian natives, their narratives of the lockdown presented an overarching theme of dejection, which was less present in Italian migrants' experiences and nearly absent in those of the Swiss natives in our sample.

#### 5.2.2 Wellbeing after 2 years of the COVID-19 pandemic: the role of social contacts

Notwithstanding the different narratives of wellbeing among the three groups, the previous sections indicate that everyone inevitably experienced a decrease in physical social contacts resulting from the COVID-19 containment policies. Given the importance of social networks for individuals' wellbeing (Helliwell and Putnam, 2004; Elgar et al., 2011; Amati et al., 2018), we aimed to inquire how social distancing regulations impacted participants' perceived wellbeing in the 2 years after the onset of the pandemic.

Our interviews reveal heterogeneous responses to social distancing; nonetheless, regardless of the perceived impact on their wellbeing, most participants employed behavior-focused coping strategies aimed at reducing probability of contagion and illness. These strategies consisted of either vaccination for the participant, social distancing habits, or a combination of the two. In some cases, these strategies were successful in supporting participants' experienced wellbeing. In other cases, they preserved one's physical wellbeing at the cost of their subjective wellbeing.

In the next sections, we explore how each of the three groups was impacted by decreased social contacts, how these sentiments developed throughout the pandemic, and how participants employed the above-mentioned coping strategies at the time of interview.

##### 5.2.2.1 Experiences of Swiss natives

Just like the lockdown did not seem to negatively impact most Swiss older adults in our study, neither did the imposed social distancing measures and related decrease in social contacts. Most of them experienced a slight change of social habits, which entailed seeing friends and family less frequently during the previous 2 years in comparison to pre-COVID times. However, these changes did not have a consequential negative impact for most of our Swiss participants. Social distancing was often described as bothersome or strange, but easily managed. Martin, for example, states:

“Yeah, [the pandemic] restricts our freedom to see – as I'm a pretty tactile person, it's true that it changes me a little bit. Friends, I kiss them less. That's what affects me a little bit more – I have to be less, much less tactile than I was with everyone, to give kisses to the left and to the right. Well, it's a bit weird.” (Martin, 75, Swiss Native)

This quote represents the sentiments expressed by most Swiss natives: they were not completely unaffected, but they were able to adapt to the changes in social habits without important repercussions for their wellbeing. At the time of interview, nearly 2 years after the pandemic onset, most Swiss older adults explained their social habits were similar to their pre-pandemic habits, but they also adopted strategies to be able to fulfill their social desires while avoiding contagion or severe illness. Most Swiss participants mentioned being vaccinated and expressed the importance of listening to scientists' advice on the preventative measures to take. These strategies helped them adjust their behaviors accordingly and feel more protected. Martin, for instance, has resumed seeing friends, but only under certain self-imposed rules. He explains,

“If we see each other, we are all vaccinated. We are not safe from catching it but at least we are less likely to get sick. And then, we avoid those who don't want to be vaccinated or those who are not vaccinated.” (Martin, 75, Swiss native)

However, one Swiss participant shared the negative experiences that followed him and his wife throughout the course of the pandemic. During the lockdown, Gianni expressed being “stuck at home,” and this lack of freedom and decreased social contacts persisted until the time of the interview, 2 years later. He says,

“Now with these problems of…the danger of contagion, and so it makes us less, less mobile, less free to live, right? Basically now, even though the lockdown has not been declared, we try to go out as little as possible, not to mingle with people so we don't get infected.” (Gianni, M, 88, Swiss native)

While most Swiss older adults were able to resume their social lives by adopting behaviors to avoid illness, the social distancing measured employed by Gianni – the oldest among our Swiss participants – allow him to preserve his physical wellbeing at the cost of his subjective wellbeing.

##### 5.2.2.2 Experiences of Italian migrants in Switzerland

In comparison to older Swiss natives, the perceived impact of social distancing measures was more heterogeneous among Italian migrants. At the time of interview, only a minority of participants said they had resumed their pre-pandemic social habits, although most slowly started seeing small groups of friends again. Like in Gianni's case, for many Italian migrants, the social distancing strategies adopted to preserve their physical wellbeing had negative repercussions on their experienced wellbeing. One participant, for example, shared that the fear of contagion remained even after containment restrictions were eased, and his personal relationships suffered. He explains,

“I lost touch with friends, you couldn't get together, you couldn't go shopping, the only thing I could do was go [walk] in the forest. Then, even when the restrictions were eased, it had affected me so much that it was hard to get together. When we got together […] we had a drink and then left. There was always that fear between us.” (Giacomo, 68, M, Italian migrant)

Giacomo looks back at his life before the pandemic with melancholy, but he also elicits the importance of acceptance and reframing one's mindset to surmount the situation. He shares:

“[Before COVID-19] we used to get together on Friday nights, play cards, drink, smoke, and for 2 years we haven't done it and I don't think we're going to start again. It's difficult because people have become distrustful, we've been wounded and we're licking our wounds. Let's put it this way. You have to get over it, direct your life differently and move on. I don't want to stay at home waiting for death.” (Giacomo, 68, M).

Although some participants were wary of resuming social activities at the time of interview, most slowly started seeing friends again while continuing to employ social distancing measures. Giulia, for instance, explains:

“[Before the pandemic] maybe we went to the restaurant once a month, or once every 2 months. But that was a lot. But we haven't done this anymore, and I didn't – and we don't even feel like doing it anymore. Now if we go to a restaurant, we go at noon…and we stand outside on the terrace because we keep being careful.” (Giulia, 70, F, Italian migrant)

Despite the slow return to a social life and the continued safety measures employed, the pandemic had a long-lasting impact on the wellbeing of most Italian older migrants, as evidenced by the following excerpts:

“I feel insecure, maybe because of the pandemic, because of the war that's going on^2^ […] I feel insecure and I tell myself I don't need this […] Insecure in the sense that I say, enough of the pandemic; insecure not physically, but in the sense that it destabilizes me [mentally] […] In the sense that I used to be able to imagine the following years and now I can't.” (Sara, 78, F, Italian migrant)

“It's 2 years that I lost and that I cannot get back. […] I lost 2 years that I won't get back. I don't even know if I'll be able to – to feel better.” (Giulia, 70, F, Italian migrant).

##### 5.2.2.3 Experiences of Italian natives in Italy

The perceived impact of the social distancing measures was notably detrimental for the experienced wellbeing of Italian natives in Italy. Most cited the lack of social contacts as the primary difficulty faced throughout the pandemic. For many, the fear instilled by the pandemic prevented them from resuming their social activities at the time of interview, despite most participants being vaccinated. This engendered feelings of sadness, anxiety, and loneliness among many Italian participants, as evidence by the following quotes:

“What I dislike is not being able to have company, because I'm all about friendships, company, laughter, and I don't like loneliness. […Before the pandemic] we used to organize trips with an association, so we would spend 15 days together, and every 2 months we would meet in an institution and spend the day together, we would eat together. With girlfriends, we would go out and take a walk in the countryside when we had nice days, and so I miss all of that now.” (Martina, 84, F, Italian native)

“Now the fact of going out and putting the mask on […], continually having to disinfect your hands when you go out, when you go get groceries, having to be careful not to get too close to people, [hoping] that in stores there aren't too many people. These – this anxiety that it gives you, that as long as you are at home, it's different. But when you go out for necessities, or go to the hospital for a visit – in short, it's anxiety, that's it. You try to – every person you meet seems to be an enemy.” (Rosa, 72, F, Italian Native)

“I have a lot of fear, really a lot, and this has prevented me from going out and also from having a social life. My social life has almost disappeared, because partly the fear, partly my age, and so the result is that while before I used to go to concerts, I used to go to the movies, now we have – my husband and I – we have canceled everything, we don't go anymore, and so there is a lot of sadness.” (Alice, 75, F, Italian native)

Although some expressed feeling safer due to the vaccine, the fear induced by the virus was still present 2 years following the pandemic onset. Many Italian natives described the continued use of their phones to communicate with friends and family – more so than during pre-pandemic times – and this kept them company. Nonetheless, most expressed that while at the beginning they tried to accept the circumstances, the pandemic had started to weigh on them and negatively influence their wellbeing. Only one Italian native shared that the changes in social habits did not have a substantial impact on his wellbeing:

“[The pandemic] did not substantially change my life, nor my family's. Of course, there were occasions when we would have liked – during the holidays, for example – to spend more time with friends. We gave this up, and we think and hope that it was accepted by our friends. In any case, this withdrawal was nothing out the ordinary, so it was nothing irrational. Let's say that it did not affect our life, our wellbeing.” (Lorenzo, 74, M, Italian native)

Yet, even for a person like Lorenzo who estimates that his wellbeing was not lowered by the pandemic, his social habits have changed, which was observed for most of the Italian natives in Italy.

## 6 Discussion

The objective of this study was to provide insight into older adults' experiences of wellbeing as well as the coping strategies employed to overcome difficulties brought about by the pandemic, in particular social distancing. Our contribution to the existing literature is 4-fold: (1) we explored older adults' lived experiences not only through their recollection of the first months of the pandemic, but also through their narratives of wellbeing and coping 2 years after the pandemic onset, (2) we analyzed the experiences of older migrants, an underrepresented population in wellbeing and COVID-19-related research, (3) we compared the experiences of two groups – Swiss natives and Italian migrants – who lived the pandemic in the same context, and (4) we compared the experiences of older adults who were subject to strict containment measures – as was the case of Italian natives – to those of adults who benefitted from more lax restrictions.

The following section discusses the results of the qualitative interviews, as well as the study limitations and implications for future policy.

While many of our interviews highlight the negative consequences of the pandemic for older adults' wellbeing in Switzerland and Italy, they also emphasize the heterogeneity of older individuals' experiences, as well as their ability to adapt and cope with stressful situations. Swiss natives and Italian migrants lived the pandemic in the same context, one that did not impose strong stay-at-home order and allowed for a certain freedom of movement. Yet, we found pronounced differences in their descriptions of wellbeing, both in the narratives concerning the first lockdown in 2020, and in the narratives addressing the following years, until time of interview.

Most Swiss natives presented positive accounts of the lockdown period; their descriptions were often coupled with coping strategies they employed to address the COVID-19 containment measures. Consistently with previous studies on coping during the pandemic, in the first months of the pandemic Swiss older adults relied on hobbies to keep busy, closeness to nature, acceptance of the sanitary situation, and cognitive strategies to find the silver lining of living through a world-wide crisis (Finlay et al., 2021; Fuller and Huseth-Zosel, 2021; Whitehead and Torossian, 2021; Brooks et al., 2022; Bustamante et al., 2022; Mau et al., 2022). Most participants described their wellbeing as unaffected even at the time of interview, 2 years after the pandemic onset. Although they described the inevitable decrease in physical contacts as bothersome, most were able to adopt behavioral strategies that involved vaccination and continued social distancing measures that kept them safe while fulfilling their social needs.

Even though Italian migrants experienced the pandemic in the same context as Swiss natives, their accounts of the lockdown and the following years were more heterogeneous. During the first months of the pandemic, they used coping strategies like those of the Swiss natives: they spent their time in nature, kept busy through hobbies, and they, too, positively referred to the freedom they felt due to Switzerland's relaxed containment measures. At this time, only some participants expressed feelings of sadness and loneliness. However, when reflecting on the entirety of the previous 2 years, most participants shared the negative impact of the pandemic on their wellbeing. Although many slowly resumed social activities at the time of interview, they evoked a continued sense of fear, distrust, and dejection. Many of their interviews demonstrated that the social distancing behaviors that allowed them to keep themselves physically safe diminished their wellbeing.

Due to the qualitative nature of this article, it is not possible to firmly assert that the different experiences of wellbeing among Swiss natives and Italian migrants are due to inequalities in reserves. However, we can posit that, at least for some Italian migrants in Switzerland, their ability to cope with the pandemic may have been partly influenced by their lower level of reserves in comparison to those of Swiss natives.

Most Italian migrants in our study migrated to Switzerland in the 1960s and 1970s, as part of the wave of labor migrants who moved from regions of Italy that lacked economic opportunities (Bolzman and Vagni, 2018; Dones and Ciobanu, 2022). Quantitative studies have revealed that, compared to older Swiss natives, older Italian migrants in Switzerland have lower education levels, report themselves in worse health, and generally occupied lower-skilled jobs (Bolzman and Vagni, 2018). For many, the migration to Switzerland as labor workers was followed by a lack of opportunities to improve their socio-economic circumstances, leaving them in worse situations in comparison to their Swiss counterparts. These disadvantaged conditions may have engendered psychological stresses that may have accumulated over the life course (Dannefer, 2003; Settersten et al., 2020), thereby impacting migrants' ability to build the adequate reserves to successfully cope with life shocks.

In our qualitative sample of Italian migrants there is an overrepresentation of highly educated participants and of participants in a comfortable financial situation, as represented by the measure “making ends meet” in Table 1 (Dones, 2023). However, on average they still have lower education levels than Swiss natives. Moreover, independently of current socioeconomic status, most participants spoke of the poverty and lack of jobs they experienced during their youth in Italy, which ultimately led them to migrate. In addition, when reflecting on other hardships encountered during their lifetimes, most cited the difficulties encountered when they migrated: discrimination, having to learn another language, detachment from family in Italy, and getting accustomed to a foreign country. Along with the disadvantaged socioeconomic conditions some participants experienced throughout the lifespan, most experienced migration-related stressors that, accumulated over the life course, may have impacted their capacity to cope with life shocks and with the pandemic in the same way that Swiss natives did. Moreover, the capacity to act in old age is dependent on the life course and the accumulation of reserves (Settersten et al., 2020), making in this case a difference between the older Swiss and older migrants.

Although Italian migrants did employ similar coping mechanisms, for most, these coping strategies were not successful in combatting the negative impact of the pandemic on their experienced wellbeing. This finding is in line with research by Pan et al. (2021), which revealed that coping strategies like increased telephone contact and increased participation in individual activities did not protect older Chinese migrants against loneliness.

Another possible explanation for the lower wellbeing expressed by Italian migrants compared to Swiss natives relates to transnational practices and attachment to the home country. Although participants did not mention their attachment to Italy when recounting their pandemic experiences, some did share the negative impact the Italian situation had on their wellbeing. Previous research stemming from the TransAge project has revealed that greater attachment to Italy correlates to greater worry about the COVID-19 pandemic (Ludwig-Dehm et al., 2023), which may have thereby impacted Italian migrants' lived experiences. Similarly, we found one case of transnational attachment among Swiss natives. The ties to Barcelona led François to value the confinement situation in Switzerland.

In comparison to older adults residing in Switzerland, older Italian natives expressed more negative emotions and difficulties when describing both the first COVID-19 lockdown and the subsequent years. Most adopted coping strategies like acceptance and increased telephone use for social contact, but the fear brought about by the virus followed them until the time of interview. This prevented most from resuming social activities, despite being vaccinated, and many expressed continued feelings of sadness, loneliness, and anxiety.

When considering the particularly negative experiences of Italian natives in Italy, we cannot propose that these were related to the various types of reserves accumulated through life, as our participants led heterogeneous life-courses. Indeed, there may be a variety of influencing factors that have the potential to affect the wellbeing of older Italian adults. One of these factors could hypothetically relate to the strict confinement measures employed by the Italian government throughout the first 2 years of the pandemic. Research thus far has revealed that countries' stringency of physical distancing regulations was associated with higher incidence of loneliness and depression among older adults (Atzendorf and Gruber, 2022; Mendez-Lopez et al., 2022). Additionally, a study on older adults in Italy showed that restrictive measures significantly impacted the quality of life, psychological wellbeing, and mobility of older adults (Tosato et al., 2022). Although no studies have yet been published on the long-term consequences of strict containment measures, our exploratory results could point to the negative impact of such regulations on older adults' experiences of wellbeing. However, this is simply a theoretical proposition and further studies on the subject are needed to firmly establish a correlation between stringency of confinement regulations and wellbeing.

Moreover, Italian natives relied on telephone communication as a coping mechanism more than the other two groups. While staying in touch with family and friends through phone and other media use has been correlated with life satisfaction during the first semi-lockdown in Switzerland (Dones et al., 2022), studies found that non-personal communication does not substitute face-to-face interactions and it is not a protective strategy against loneliness among older adults (Pan et al., 2021; König and Isengard, 2023). Further research should thus address the effectiveness of different coping strategies in times of crisis.

### 6.1 Limitations, strengths, and suggestions for future research

This study does not come without limitations. Due to the qualitative nature of the research and the relatively small sample size, results cannot be generalized even though saturation of responses was reached. In addition, our study did not explore the experiences of many people who lived alone during the pandemic, a population that might have been particularly at risk of social isolation. Similarly, there is a possibility that older adults with lower levels of wellbeing may not have been willing to participate to the research, although some research participants shared their difficulties and negative experiences of the pandemic. Lastly, to be able to better understand the role of reserves in older adults' experiences of the pandemic, longitudinal, quantitative data would be necessary.

Nonetheless, this article sheds light on several aspects. First, despite the homogeneous representation of older adults as frail and vulnerable (Petretto and Pili, 2020; Ayalon et al., 2021; Maggiori et al., 2022), the pandemic impact on wellbeing is not the same for all older adults, as demonstrated by emerging studies (Wettstein et al., 2022a,b) and by the different experiences of this article's older populations. Second, despite the employment of coping strategies used by all participants, their effectiveness in mediating the long-term impact of the pandemic on experiences of wellbeing differed among groups. Third, the long-term impact of the pandemic and the various containment strategies needs further examination. As the case of Italian migrants in Switzerland shows, some older migrants experienced the beginning of the pandemic in quite positive ways, but their narratives of their situation 2 years after the pandemic onset showed an overall negative effect on their wellbeing.

The share of older adults in Europe continues to increase (Eurostat, 2023), as does the share of older migrants (UNDESA, 2020). The advancements of the last few decades have reduced the dependence of older adults and have increased life expectancy. At the same time, social inequalities and inter-individual diversity make of today's older adults an increasingly heterogeneous group (Oris et al., 2020). The consideration of this heterogeneity should be at the core of not only scientific research, but also of policy interventions, as grouping all older adults under the “vulnerable and frail” umbrella propagates against narratives that can lead to increased psychological distress and negative self-perceptions of aging (Losada-Baltar et al., 2021; Derrer-Merk et al., 2022a,b).

To account for the diversity in older adults' lives, research on the long-term impact of the pandemic should adopt a life-course approach to further analyze how differing trajectories engender situations of resilience or vulnerability. Given the increase of share of older migrants, their underrepresentation in COVID-19 and wellbeing research, and the possible long-term effects of having a migration background, special consideration should be allotted to them. Moreover, studies should further address the effectiveness of coping strategies among different populations. Lastly, in cases of future health crises, governments should have an increased regard for the negative consequences of stringent confinement measures, as social isolation and physical inactivity among older adults are correlated with increased hospitalization, depression, cognitive impairment, and reduced quality of life (Cacioppo et al., 2010; Cacioppo and Cacioppo, 2014; Ozemek et al., 2019).

## Data availability statement

The datasets presented in this article are not readily available because the qualitative interviews analyzed in this study are not publicly available. For now, they are available from RC on reasonable request. Requests to access the datasets should be directed to RC, oana.ciobanu@hetsl.ch.

## Ethics statement

The studies involving humans were approved by the Ethics Committee of the Faculty of Social Sciences of the University of Geneva. The studies were conducted in accordance with the local legislation and institutional requirements. Written informed consent for participation was not required from the participants or the participants' legal guardians/next of kin because participation in the study was voluntary, and all participants gave oral consent to be interviewed and recorded.

## Author contributions

ID drafted the interview guidelines, carried out the data collection and analysis, and was the major contributor in writing the manuscript. RC supervised the project, reviewed and approved the interview guidelines, provided article references, read parts of the interviews, and contributed to the discussion and conclusion. All authors read and approved the final manuscript.

## Footnotes

^1^Of these phone interviews, one was done through WhatsApp audio, the rest through the regular phone line.

^2^The participant is referring to the war between Russia and Ukraine, which had just begun at the time of interview.
